# The Role of Everyday Executive Function in Observed Social Symptoms of Autism Spectrum Disorder

**DOI:** 10.1007/s10803-024-06351-0

**Published:** 2024-05-03

**Authors:** Christina Burroughs, Rachael A. Muscatello, Blythe A. Corbett

**Affiliations:** 1 Department of Psychiatry and Behavioral Sciences, Vanderbilt University Medical Center, 1500 21st Avenue South, Nashville, TN 37212 USA; 2 Vanderbilt University Medical Center, Vanderbilt Kennedy Center, Nashville, TN USA; 3 Department of Psychology, Vanderbilt University, Nashville, TN USA

**Keywords:** Autism, Executive function, Sex differences, Camouflaging, Social impairment

## Abstract

Recent research suggests there may be differences in the social presentations of autistic males and females. Camouflaging is believed to account for some of these differences and executive function (EF) may support compensatory social behaviors. As few studies have explored the role of sex and everyday EF when evaluating specific social difficulties among autistic youth, the present study seeks to address this. The Social Responsiveness Scale-2 (SRS-2) was used to measure types of social difficulties and the Behavioral Rating Inventory of Executive Function-2 (BRIEF-2) served as a measure of everyday EF. Four three-step hierarchical multiple regressions were conducted with SRS-2 social subscales as dependent variables. Autism symptom severity, BRIEF-2 EF Indices (i.e., behavioral, emotional, and cognitive regulation), and sex served as independent variables. Types of EF impairment significantly predicted social symptoms of autism. Behavioral dysregulation predicted all social symptoms assessed, cognitive dysregulation predicted social awareness and communication challenges, and emotion dysregulation predicted social motivation and communication difficulties. Sex significantly predicted social communication and cognition challenges, beyond the contributions of age, IQ, autism severity, and EF impairment. Findings from this study provide evidence for the contribution of EF to observed social symptoms of autism. Results suggest there may be sex-based differences in the relationship between EF and social problems for autistic youth. Implications and future directions are discussed.

## Introduction

Autism spectrum disorder (ASD) is a heterogeneous neurodevelopmental disorder defined by social communication challenges and repetitive and restricted behaviors and interests (American Psychiatric Association, 2013). Factors such as age, intellectual ability, and co-occurring psychiatric conditions are known to impact the presentation of autistic symptoms (Masi et al., 2017), and a growing body of research points to the role of biological sex in divergent symptom presentations (de Giambattista et al., 2021; Wood-Downie et al., 2021a). Historically, autism has been considered a predominately male disorder with a commonly reported male-to-female sex ratio of 4:1 (Baio et al., 2018), though recent findings suggest this ratio may be closer to 3:1 or 2:1 (D’Mello et al., 2022; Loomes et al., 2017). Results from studies with increased female representation and varied symptom assessments suggest there are developmental, quantitative, and qualitative differences in the presentations of autistic males and females, particularly related to social communication (Lai et al., 2015; McFayden et al., 2023; Wood-Downie et al., 2021a). It has been proposed that there is a distinct female autism phenotype (Lai et al., 2015) characterized by higher levels of camouflaging behaviors (i.e., strategies to mask autistic symptoms to navigate social interactions more smoothly) and the appearance of milder social communication impairments (Corbett et al., 2021; Hull et al., 2020a, 2020b; Wood-Downie et al., 2021a). As camouflaging is believed to involve conscious and unconscious strategies to appear ‘less autistic’ in social interactions, executive function (EF) abilities may support compensatory social behaviors. As few studies have explored the role of both sex and everyday EF when evaluating specific social communication impairments among youth with ASD, the present study seeks to address this.

### Sex Differences in Social Symptoms of ASD

A burgeoning body of research suggests there may be important differences in the social presentations of males and females with ASD that have implications for diagnostic and treatment efforts. As autism diagnostic criteria and tools were largely developed based on a male-typical phenotype, females without severe symptoms and co-occurring conditions (e.g., intellectual disability) may be less likely to receive a timely ASD diagnosis (Loomes et al., 2017). As such, the majority of studies employing broad construct measures (i.e., diagnostic criteria, gold-standard diagnostic instruments) have found no sex differences in social communication and interaction among autistic youth (see meta-analyses Hull et al., 2017a; Mahendiran et al., 2019; Van Wijngaarden-Cremers et al., 2014). As diagnostic instruments may not be sensitive to the way ASD manifests in females, it has been proposed that narrow construct measures may better elucidate potential social sex differences (Hull & Mandy, 2017; Lai & Szatmari, 2020). In support of this, a recent meta-analysis synthesized findings across 16 studies employing narrow social and communication construct measures (e.g., social attention, peer relationships, social reciprocity) and found that autistic females demonstrated significantly better social interaction and communication skills compared to autistic males (Wood-Downie, et al., 2021a). Findings support the notion that autistic females are better able to camouflage social communication difficulties and present as more socially advanced than autistic males in certain contexts (Hull et al., 2020a, 2020b; Wood-Downie et al., 2021b).

The concept of camouflaging in the context of sex differences in ASD has gained increased attention in recent years. It is broadly defined as the employment of specific behavioral and cognitive strategies by autistic people to adapt to or cope in a predominately non-autistic social world (Cook et al., 2022; Hull et al., 2017b; Livingston & Happé, 2017). Camouflaging may enable an individual to consciously or unconsciously present as non-autistic by masking autistic behaviors and/or employing compensatory strategies to overcome social difficulties (Lawson, 2020; Livingston et al., 2019). Camouflaging strategies may include forcing eye contact, using conversational scripts, mimicking nonverbal communication, and applying learned social rules to respond to others' verbal and nonverbal communication (Livingston & Happé, 2017). While autistic individuals may engage in compensatory social behaviors via cognitive and behavioral strategies, the underlying social-cognitive difficulties inherent in ASD may remain under the surface. Camouflaging has been proposed as an explanation for missed or late ASD diagnosis in females, and is considered a key mechanism underlying the female autism phenotype (Hull et al., 2020a, 2020b; Lai et al., 2015). Several studies have found that autistic females engage in greater camouflaging compared to males (Hull et al., 2020a, 2020b; Lai et al., 2017; Wood-Downie et al., 2021b).

Executive function (EF) is an umbrella term referring to various mental operations (e.g., inhibition, set-shifting, flexibility, planning/organization, self-monitoring, working memory) that collectively enable an individual to respond adaptively and engage in goal-directed, purposeful behaviors (Lezak, 2012). EF abilities may facilitate camouflaging and compensatory social behaviors among youth with ASD (Hull et al., 2021; Livingston & Happé, 2017). For example, greater EF may enable self-monitoring and inhibition of autistic behaviors, planning of social scripts, shifting attention to social cues, and flexible adaptation of social responses within different contexts. As EF abilities may support camouflaging behaviors in autistic youth and differentially impact the presentation of social symptoms for males and females, there remains a need to understand how sex is related to EF and social symptoms of ASD.

### EF, Social Symptoms, and Sex in ASD

Research on EF in ASD supports the existence of broad executive dysfunction (see Demetriou et al., 2018 for review) that contributes to social impairment (Freeman et al., 2017; Kenworthy et al., 2009; Leung et al., 2016). Studies investigating sex differences in EF domains using performance-based tasks have yielded mixed findings and no significant sex differences have been observed across studies using parent-report behavioral measures assessing EF in everyday situations (Demetriou et al., 2018). Research on EF in ASD has shifted in recent years to measuring everyday EF via parent-report measures rather than performance-based measures, which sample fractioned EF skills that may not translate into everyday functioning (Demetriou et al., 2018; Leung et al., 2016). Everyday EF measures, such as the Behavioral Rating Inventory of Executive Function (BRIEF/BRIEF-2; Gioia et al., 2000, 2015), demonstrate superior ecological validity and clinical utility (Demetriou et al., 2018). As such, studies employing the BRIEF to investigate everyday EF in autistic youth are summarized.

Initial studies established the relationship between EF and social functioning among autistic youth. Kenworthy et al. (2009) found that BRIEF behavioral regulation (i.e., inhibition, shifting, emotional control) and metacognitive EF (i.e., planning/organization, organization of materials, initiation, working memory, monitoring) were correlated with reciprocal social interaction challenges on the Autism Diagnostic Interview (ADI/ADI-R; Le Couteur et al., 1989; Lord et al., 1994) and Autism Diagnostic Observation Schedule (Lord et al., 1999) among 89 autistic youth. Leung et al. (2016) found that BRIEF behavioral regulation and metacognitive EF predicted global social communication impairment on the Social Responsiveness Scale (SRS; Constantino & Gruber, 2005) among 70 autistic youth aged 6–15. Further, Torske et al. (2018) investigated associations among the BRIEF indices and SRS subscales and found that metacognitive EF was significantly associated with all social subscales (e.g., social communication, social cognition, social awareness, social motivation), while behavioral regulation was associated with all but social motivation among 86 autistic youth 6–18. Together, these studies suggest both behavioral regulation and metacognitive EF contribute to social difficulties for autistic youth.

A handful of studies expanded on these findings and additionally explored the role of sex. Chouinard et al. (2019) found that metacognitive EF significantly predicted the SRS Social Communication Index score beyond the contributions of behavioral regulation and demographic factors (e.g., age, IQ) among 117 youth 5–18 with ASD. Results suggested a different relationship between EF and social communication for males and females, as behavioral regulation was a significant predictor of social impairment for males, but not for females. However, the study was underpowered to further examine sex differences in this relationship. Torske et al. (2018) investigated the potential moderating role of sex in the relationship between global EF difficulties on the BRIEF Global Executive Composite and autistic symptoms on ADI-R domains among 116 (25 females, 91 males) aged 5–19. A significant sex-specific relationship between global EF challenges and reciprocal social interaction and communication was found, with females showing a stronger relationship between EF and social symptoms of ASD. Lastly, Bednarz et al. (2020) investigated the relationship between BRIEF EF problems and social impairment on the ADOS-2 (Lord et al., 2012), ADI-R, and SRS among 106 (85 males, 21 females) autistic youth 5–13. While BRIEF behavior regulation and metacognitive indices were not associated with social impairment on the ADOS-2 or ADI-R after accounting for age, IQ, and sex, significant relationships between EF and social symptoms were observed on the SRS. Metacognitive EF and behavior regulation were significantly associated with the SRS Total score; female sex was associated with a stronger relationship between metacognitive EF and the SRS total, consistent with results from Chouinard et al. (2019). BRIEF metacognitive EF and behavior regulation were both significant predictors of social communication and social motivation. Behavior regulation predicted social cognitive difficulties and metacognitive EF predicted problems with social awareness. Results support the idea that EF components differentially impact types of social difficulties for autistic youth and that diagnostic instruments may not be ideal for capturing these subtleties.

### Present Study

Despite interest in the associations among sex, EF, and specific social symptoms, research to date is limited warranting further research. All of the reviewed studies employed the original version of the BRIEF, consisting of metacognitive and behavior regulation indices, as measures of EF components which have been shown to predict social impairments in autistic youth (Bednarz et al., 2020; Chouinard et al., 2019; Kenworthy et al., 2009; Leung et al., 2016; Torske et al., 2018, 2022). The revised BRIEF now consists of emotional, behavioral, and cognitive regulation indices, enabling further research on the contributions of these EF constructs to social symptoms. Additionally, many of the previous studies have measured social impairment via diagnostic instruments and global measures of social impairment (e.g., composite scores, total scores), therefore it is unclear how EF difficulties relate to specific types of social challenges exhibited by autistic youth. Moreover, because social difficulties are often the primary target of clinical intervention and may present differently in males and females, the present study seeks to better understand the role of EF in observed social impairments.

The aim of the study was to explore the associations between sex (female, male), specific indices of EF impairment (behavioral, cognitive, and emotional regulation), and types of social impairments (social awareness, cognition, motivation, communication) for youth with ASD within a hierarchical regression framework. It was hypothesized that executive functioning impairments would significantly predict all social symptoms assessed. Sex was hypothesized to be a significant predictor of all social symptoms, beyond the effect of EF. As the factor structure of the BRIEF-2 (Gioia et al., 2015) utilized in the current study differs from that of the BRIEF employed in previous studies, the investigation of the contribution of BRIEF-2 indices was exploratory in nature.

## Methods

### Participants and Procedures

Data were utilized from a previously completed longitudinal study focused on pubertal development (MH111599 PI: Corbett). The current study includes data from the initial year of enrollment when participants were between 10 years, 0 months, and 13 years, 11 months of age. The total sample included 140 early adolescents with ASD, including 104 males and 36 females (*M* = 11.43, *SD* = 1.03). All participants had a diagnosis of ASD confirmed for eligibility by licensed psychologist with autism expertise based on criteria set forth in the Diagnostics and Statistical Manual, Fifth Edition (DSM-5; American Psychiatric Association, 2013) and a score at or above the clinical cut-off on the Autism Diagnostic Observation Schedule, Second Edition (ADOS-2; Lord et al., 2012) and a full-scale IQ of 70 or higher on the Wechsler Abbreviated Scale of Intelligence–Second Edition (Wechsler, 2011).

## Measures

### Clinical Characterization

Parents of participants provided demographic information, including the child’s age, gender, race, ethnicity, and household income. A Full-Scale IQ score was obtained for all participants. The ADOS-2 Calibrated Severity Score (ADOS CSS; Esler et al., 2015; Gotham et al., 2009; Hus & Lord, 2014) served as a measure of autism symptom severity. The ADOS CSS is based on a 10-point scale: scores from 1 to 3 are in the ‘‘Nonspectrum’’ range; 4–5 are in the ‘‘ASD’’ range; and 6–10 in the ‘‘Autism’’ range. The CSS severity metric was found to have more uniform distributions across developmental groups than raw scores and to be less influenced by child characteristics such as verbal IQ, nonverbal IQ, age, and race (Gotham et al., 2009; Hus & Lord, 2014).

### Executive Functioning

The Behavior Rating Inventory of Executive Function, Second Edition (BRIEF-2; Gioia et al., 2015) parent-report questionnaire was employed to assess behavioral manifestation of EF problems. Development of the BRIEF-2 was informed by factor analytic research on the original BRIEF that supported reorganization of the eight scales into nine by splitting the Monitor scale into Task-Monitor and Self-Monitor, and superior fit for a three-factor solution (Gioia et al., 2002). The original BRIEF contained 86 items designed to measure eight EF constructs (i.e., scales) that comprise the Metacognitive Index (Plan/Organize, Working Memory, Initiate, Organization of Materials, and Monitor scales) and the Behavior Regulation Index (Inhibit, Shift, Emotional Control scales). The BRIEF-2 contains 63 items with nine clinical scales that combine to form the Global Executive Composite (GEC) and three following index scores used in the present study: Behavior Regulation Index (BRI; comprised of the Inhibit and Self-Monitor domains), Emotional Recognition Index (ERI; comprised of the Shift and Emotional Control domains), and Cognitive Regulation Index (CRI; comprised of the Initiate, Working Memory, Plan/Organize, Task-Monitor, and Organization of Materials domains). The BRIEF-2 index scores have robust psychometric properties (Gioia et al., 2015), including excellent internal consistency (α = 0.90–0.97) and good test–retest reliability (BRI = 0.83, ERI = 0.82, CRI = 0.89). Index scores are reported as T-scores, with higher scores indicating more impairment and scores ≥ 65 falling in the clinically elevated range.

### Social Impairment

The Social Responsiveness Scale (SRS-2; Constantino & Gruber, 2012) served as a parent-report measure of social functioning. The SRS-2 is a 65-item questionnaire that generates an overall Total T-Score of autism symptom severity, and the following social subscales: Social Awareness, Social Cognition, Social Communication, and Social Motivation.* T*-scores ≥ 76 indicate severe deficits related to ASD that significantly impact interactions with others. Scores 66–75 indicate moderate difficulties; scores 60–65 indicate mild difficulties; and scores of ≤ 59 suggest that the examinee does not demonstrate impairment consistent with an ASD diagnosis. The SRS-2 has good construct validity (Constantino et al., 2003) and strong reliability (Constantino & Gruber, 2005), with a coefficient alpha of 0.95.

### Analytic Plan

Data were cleaned and preliminary analyses were conducted to ensure no violations of normality, linearity, and homoscedasticity assumptions using SPSS Statistics Software, Version 28. ﻿Homoscedasticity was evaluated by visually inspecting scatterplots of the predicted values and residuals for random distribution above and below zero on both x- and y-axes. Multicollinearity was evaluated by checking the variance inflation factor (VIF) to determine that they were < 10 (acceptable) or < 5 (ideal). Normality was evaluated by visually inspecting whether residuals fell along the diagonal line of the respective P-P Plots. All assumptions were met.

Descriptive statistics were generated, and bivariate analyses were performed to compare males and females on demographic and clinical characteristics (See Table 1). Statistically significant differences in demographic and clinical characteristics were examined with Fisher’s exact test, chi-square test, likelihood ratio test, or t-test depending on the type of variable (i.e., categorical or continuous) and cell size. While 140 participants had demographic and clinical data reported in Table 1, there was some missing data on measures of social and executive functioning detailed in Table 2.

**Table 1:** Descriptive statistics and group comparisons on demographic and clinical characteristics

	Total	Males	Females	Sig. dif
	*n* (%)/*M *(*SD*)			
*n*	140	104	36	
ADOS CSS	7.21 (1.92)	7.43 (1.87)	6.54 (1.95)	*t*(138) = 2.41, *p* = 0.017
Age	11.43 (1.03)	11.47 (1.03)	11.32 (1.05)	*t*(138) = 0.75, *p* = 0.46
Ethnicity				
Non-Hispanic	129 (92.14%)	98 (94.23%)	31(86.11%)	*p* = 0.117
Hispanic	11 (7.86%)	6 (5.77%)	5 (13.89%)	
Household income				
< 25,000	14 (10.0%)	11 (10.6%)	3 (8.3%)	χ^2^ (8) = 13.83, *p* = 0.09
25–50,000	28 (20.0%)	19 (18.3%)	9 (25.0%)	
50–75,000	23 (16.4%)	19 (18.3%)	4 (11.1%)	
75–100,000	17 (12.1%)	16 (15.4%)	1 (2.8%)	
100–150,000	21 (15.0%)	16 (15.4%)	5 (13.9%)	
> 150,000	16 (11.4%)	7 (6.7%)	9 (25.0%)	
Not reported	21 (15.0%)	16 (15.4%)	5 (13.9%)	
IQ	101.04 (20.65)	101.54 (21.09)	99.61 (19.56)	*t*(138) = 0.48, *p* = 0.62
Race				
White	114 (81.4%)	85 (81.7%)	29 (80.6%)	χ^2^ (3) = 3.69, *p* = 0.30
Black	17 (12.1%)	12 (11.5%)	5 (13.9%)	
Asian	1 (0.7%)	0 (0.0%)	1 (2.8%)	
Multiracial	8 (5.7%)	7 (6.7%)	1 (2.8%)	

The ADOS-SA-CSS is a severity metric with a possible range from 1 to 10, with higher scores indicating greater impairment

**Table 2:** Descriptive statistics for males and females on measures of executive function and social functioning

	Males *M* (*SD*)	Females *M* (*SD*)
*n*	102	35
BRIEF-2 indices		
BRI	67.57 (10.24)	69.03 (11.52)
ERI	73.08 (10.48)	71.56 (12.83)
CRI	68.25 (8.44)	66.69 (11.38)
*n*	104	36
SRS-2 subscales		
Social awareness	70.78 (10.67)	70.14 (10.91)
Social cognition	70.75 (11.46)	74.75 (9.99)
Social communication	72.80 (10.60)	77.67 (10.01)
Social motivation	67.61 (11.92)	70.33 (9.45)

*BRI* BRIEF-2 Behavior Regulation Index, *ERI* BRIEF-2 Emotion Regulation Index, *CRI* BRIEF-2 Cognitive Regulation Index

Four three-step hierarchical multiple regressions were conducted with the SRS-2 social awareness, social cognition, social communication, and social motivation subscale T scores as dependent variables. Independent variables included autism symptom severity (i.e., ADOS CSS), measures of executive functioning (i.e., BRIEF-2 behavior regulation, emotion regulation, cognitive regulation index scores), and sex (Tables 3, 4, 5, 6). Autism symptom severity was entered in step 1, measures of executive functioning were entered in step 2, and sex was entered in step 3.

**Table 3:** Hierarchical multiple regression analyses for social awareness

	*B (SE)*	β	*R*^*2*^	*ΔR*^*2*^	*p*
Step 1			0.00		0.872
ADOS CSS	− 0.08 (0.49)	− 0.01			0.872
Step 2				0.36	< .001
ADOS CSS	0.41 (0.40)	0.07			0.308
BRI	0.36 (0.11)	0.35			0.002
ERI	0.07 (0.09)	0.08			0.431
CRI	0.32 (0.11)	0.27			0.003
Step 3				0.00	0.999
ADOS CSS	0.41 (0.41)	0.07			0.321
BRI	0.36 (0.11)	0.35			0.002
ERI	0.07 (0.10)	0.08			0.435
CRI	0.32 (0.11)	0.27			0.003
Sex	− 0.002 (1.84)	0.00			0.999

*ADOS CSS* ADOS-2 Calibrated Severity Score, *BRI* BRIEF-2 Behavior Regulation Index, *ERI* BRIEF-2 Emotion Regulation Index, *CRI* BRIEF-2 Cognitive Regulation Index, *B* (*SE*) unstandardized beta (standard error for the unstandardized beta), *β* standardized beta, *R*^2^ coefficient of determination, Δ*R*^2^ change in the coefficient of determination, *p* probability value

**Table 4:** Hierarchical multiple regression analyses for social cognition

	*B (SE)*	β	*R*^*2*^	*ΔR*^*2*^	*p*
Step 1			0.001		0.782
ADOS CSS	− 0.14 (0.50)	− 0.02			0.782
Step 2				0.273	< .001
ADOS CSS	0.33 (0.44)	0.06			0.452
BRI	0.39 (0.12)	0.37			0.002
ERI	0.13 (0.10)	0.13			0.207
CRI	0.11 (0.12)	0.09			0.327
Step 3				0.024	< .001
ADOS CSS	0.52 (0.44)	0.09			0.237
BRI	0.35 (0.12)	0.33			0.005
ERI	0.16 (0.10)	0.15			0.133
CRI	0.15 (0.12)	0.12			0.200
Sex	4.21 (1.99)	0.16			0.037

*ADOS CSS* ADOS-2 Calibrated Severity Score, *BRI* BRIEF-2 Behavior Regulation Index, *ERI* BRIEF-2 Emotion Regulation Index, *CRI* BRIEF-2 Cognitive Regulation Index, *B* (*SE*): unstandardized beta (standard error for the unstandardized beta), *β* standardized beta, *R*^2^ coefficient of determination, Δ*R*^2^ change in the coefficient of determination, *p* probability value

**Table 5:** Hierarchical multiple regression analyses for social communication

	*B (SE)*	β	*R*^*2*^	*ΔR*^*2*^	*p*
Step 1			0.00		0.907
ADOS CSS	− 0.56 (0.47)	− 0.10			0.907
Step 2				0.402	< .001
ADOS CSS	0.45 (0.38)	0.08			0.235
BRI	0.35 (0.11)	0.35			0.001
ERI	0.20 (0.09)	0.21			0.029
CRI	0.22 (0.10)	0.19			0.032
Step 3				0.048	0.001
ADOS CSS	0.71 (0.37)	0.13			0.058
BRI	0.30 (0.10)	0.30			0.005
ERI	0.23 (0.09)	0.24			0.009
CRI	0.26 (0.10)	0.23			0.008
Sex	5.63 (1.67)	0.23			0.001

*ADOS CSS* ADOS-2 Calibrated Severity Score, *BRI* BRIEF-2 Behavior Regulation Index, *ERI* BRIEF-2 Emotion Regulation Index, *CRI* BRIEF-2 Cognitive Regulation Index, *B* (*SE*): unstandardized beta (standard error for the unstandardized beta), *β* standardized beta, *R*^2^ coefficient of determination, Δ*R*^2^ change in the coefficient of determination, *p* probability value

**Table 6:** Hierarchical multiple regression analyses for social motivation

	*B *(*SE*)	β	*R*^*2*^	*ΔR*^*2*^	*p*
Step 1			0.00		0.923
ADOS CSS	0.05 (0.51)	0.01			0.923
Step 2				0.187	< .001
ADOS CSS	0.16 (0.47)	0.03			0.730
BRI	− 0.25 (0.13)	− 0.23			0.063
ERI	0.51 (0.11)	0.49			< .001
CRI	0.18 (0.12)	0.15			0.154
Step 3				0.023	0.057
ADOS CSS	0.35 (0.48)	0.06			0.459
BRI	− 0.29 (0.13)	− 0.26			0.034
ERI	0.53 (0.11)	0.52			< .001
CRI	0.21 (0.12)	0.17			0.090
Sex	4.14 (2.15)	0.16			0.057

*ADOS CSS* ADOS-2 Calibrated Severity Score, *BRI* BRIEF-2 Behavior Regulation Index, *ERI* BRIEF-2 Emotion Regulation Index, *CRI* BRIEF-2 Cognitive Regulation Index, *B* (*SE*) unstandardized beta (standard error for the unstandardized beta), *β* standardized beta, *R*^2^ coefficient of determination, Δ*R*^2^ change in the coefficient of determination, *p* probability value

## Results

Bivariate analyses revealed no significant differences between males and females on age, IQ, race, ethnicity, parental education, or household income.

### Social Awareness

The hierarchical multiple regression revealed that at step 1, autism symptom severity did not significantly contribute to the model, *F*(1,133) = 0.03, *p* = 0.87. Introducing the BRIEF-2 indices at step 2 (e.g., behavior regulation, emotion regulation, cognitive regulation) explained 36.30% of the variance in social awareness, and this *ΔR*^*2*^ was significant, *F*(3, 130) = 18.57, *p* < 0.001. The addition of sex at step 3 did not significantly contribute to the model (*p* = 0.99). Of the independent variables of interest, behavior regulation (β = 0.35, *p* = 0.002) and cognitive regulation (β = 0.27, *p* = 0.003) were significantly associated with social awareness.

### Social Cognition

The hierarchical multiple regression revealed that at step 1, autism symptom severity did not significantly contribute to the model, *F*(1,133) = 0.08, *p* = 0.78. Introducing the BRIEF-2 indices at step 2 explained 27.20% of the variance in social cognition, and this *ΔR*^*2*^ was significant, *F*(3, 130) = 12.18, *p* < 0.001. The addition of sex at step 3 significantly contributed to the model *F*(1,129) = 10.90, *p* < 0.001, and explained an additional 2.40% of the variance in social cognition. Of the independent variables of interest, behavior regulation (β = 0.33, *p* = 0.005) and sex (β = 0.16, *p* = 0.037) were significantly associated with social cognition.

### Social Communication

The hierarchical multiple regression revealed that at step 1, autism symptom severity did not significantly contribute to the model, *F*(1,133) = 0.01, *p* = 0.91. Introducing the BRIEF-2 indices at step 2 explained 40.20% of the variance in social communication, and this *ΔR*^*2*^ was significant, *F*(3, 130) = 21.83, *p* < 0.001. The addition of sex at step 3 significantly contributed to the model *F*(1,129) = 21.13, *p* < 0.001 and explained an additional 4.80% of the variance in social communication. Behavior regulation (β = 0.30, *p* = 0.005), emotion regulation (β = 0.24, *p* = 0.009), cognitive regulation (β = 0.23, *p* = 0.009), and sex (β = 0.23, *p* = 0.001) were significantly associated with social communication.

### Social Motivation

The hierarchical multiple regression revealed that at step 1, autism symptom severity did not significantly contribute to the model, *F*(1,133) = 0.01, *p* = 0.92. Introducing the BRIEF-2 indices at step 2 explained 18.70% of the variance in social motivation, and this *ΔR*^*2*^ was significant, *F*(3, 130) = 7.49, *p* < 0.001. The addition of sex at step 3 did not significantly contribute to the model, *F*(1,129) = 6.85, *p* = 0.057. Behavior regulation (β = -0.26, *p* = 0.034) and emotion regulation (β = 0.52, *p* < 0.001) were significantly associated with social motivation.

## Discussion

The present study explored the associations between sex (female, male), specific indices of EF impairment (behavioral, cognitive, and emotional regulation), and types of social impairments (social awareness, cognition, motivation, communication) among a well-characterized sample of youth with ASD. As hypothesized, indices of EF significantly predicted social symptoms of autism. Specifically, behavioral dysregulation was a significant predictor of all types of social impairment, including social awareness, cognition, communication, and motivation. Cognitive dysregulation predicted challenges with social awareness and social communication. Emotion dysregulation predicted problems with social motivation and social communication. Contrary to our hypothesis, sex was not significantly associated with all types of social impairment assessed. Sex was a significant predictor of social communication and social cognition, beyond the contributions of age, IQ, autism severity, and EF impairment.

Behavioral dysregulation, or problems with inhibition and self-monitoring, predicted all social difficulties assessed. Inhibition captures difficulties evaluating consequences before acting and putting the brakes on behaviors, and self-monitoring refers to awareness of how one’s behavior affects others. Problems in these areas may hinder attention to social cues to guide behavior (social awareness), interpretation of others’ communication to understand social cause and effect (social cognition), communication of ideas/feelings (social communication), and social interest and engagement (social motivation). The results seem similar to previous studies that have found the BRIEF-2 behavioral regulation problems significantly predicted difficulties with social inferencing, social knowledge (Fong & Iarocci, 2020), and verbal communication (Hutchison et al., 2020) on the Multidimensional Social Competence Scale (Yager & Iarocci, 2013) among autistic youth.

Cognitive dysregulation, or problems with initiation, working memory, planning/organization, task monitoring, and organization of materials, were significantly associated with difficulties in social awareness and social communication. Trouble focusing attention, appropriately starting and concluding social interactions, and keeping up with complex social information contributes to problems orienting to important social information and managing the demands of back-and-forth conversation (e.g., answering questions, responding to multiple pieces of information). A prior study employing the BRIEF found that the Metacognitive Index predicted SRS social awareness and social communication scores for autistic youth (Bednarz et al., 2020). Follow-up analyses showed that the monitoring scale was associated with worse social awareness and communication, and poorer working memory abilities were predictive of greater communication challenges (Bednarz et al., 2020). As there is considerable construct overlap between the BRIEF Metacognitive Index and the BRIEF-2 Cognitive Regulation Index (*r* = 0.96; Gioia et al., 2015), our results are in line with these findings.

Emotion dysregulation, or difficulties shifting attention/behavior and demonstrating emotional control, were significantly associated with problems in social communication and social motivation. Overattention to unrelated or non-social information, difficulties adjusting thinking/behavior amid change, and problems managing reactions can hinder social orientation, appropriate responding, and social confidence. A prior study found that the BRIEF Shift scale, included in its entirety within the BRIEF-2 Emotion Regulation Index, and the Flexibility Scale-Revised (Strang et al., 2017) significantly predict challenges with communication and social adaptive behavior (Bertollo et al., 2020). Our results are in line with those of Bertollo and colleagues and suggest difficulties shifting and regulating emotions amid change are associated with social communication and social engagement challenges for autistic youth.

In considering the role of sex in the relationship between everyday EF and social symptoms of ASD, sex was a significant predictor of challenges with social communication and social cognition above and beyond the role of age, IQ, autism symptom severity, and EF measures. It is interesting that females on average displayed more impairment in social communication and cognition than males. This finding is consistent with results from large-scale studies using data from the Longitudinal Study of Children with Autism (ELENA) and Simons Simplex cohorts (Dellapiazza et al., 2022; Frazier et al., 2014) and an investigation of item-level sex differences on the SRS-2 among age- and IQ-matched autistic youth (Bitsika & Sharpley, 2019). While social sex differences have not been reliably demonstrated using observational diagnostic assessments (Hull et al., 2017a; Mahendiran et al., 2019; Van Wijngaarden-Cremers et al., 2014), studies applying qualitative and computerized techniques to assess narrow constructs during ADOS-2 Module 3 activities have found that females show better social cognition and understanding of social causality (Mattern et al., 2023), superior gestural communication (Rynkiewicz et al., 2016), and increased use of social words during conversation (Cola et al., 2022).

As the SRS-2 utilizes parent report, it is possible that parents have higher social expectations for females compared to males, resulting in more severe endorsements of social problems. Additionally, females have been found to engage in greater camouflaging compared to males (Hull et al., 2020a, 2020b; Lai et al., 2017; Wood-Downie et al., 2021b), so it is possible that parents tend to report on social symptoms observed in the absence of camouflaging behaviors. This may result in greater reported social difficulties than might be evident in interactions with diagnosticians or peers. Hannon and colleagues (2023) found that neither self- or parent-reported camouflaging on the Camouflaging Autistic Traits Questionnaire (CAT-Q; Hull et al., 2019) was significantly correlated with SRS-2 social problems or camouflaging discrepancy scores (Schuck et al., 2019), derived from the differences between internal autistic characteristics (i.e., self-reported) and external presentations (i.e., ADOS calibrated severity score). This supports the idea that the construct of camouflaging is distinct from social abilities, whether rated by autistic people themselves, their parents, or clinicians (Hannon et al., 2023). Thus, females who engage in greater camouflaging behaviors may not be rated by parents as having milder social communication and interaction challenges on the SRS-2. More research employing a variety of measurement approaches is needed to better understand the relationship between camouflaging and observed social impairments from the perspectives of autistic individuals, their parents, and clinicians. Since prior studies have highlighted that clinicians and teachers may rate females as having milder social impairments than parents (Hiller et al., 2014, 2016; Ratto et al., 2018), future studies should examine how parent endorsements align with observations made by clinicians and teachers to determine the source and implications of potential discrepancies.

This study contributes to understanding of the relationship between EF constructs and specific social symptoms for autistic youth. It was a necessary first step to explore these associations using the current version of the BRIEF-2 (Gioia et al., 2015), which comprises different EF constructs than the previous version. Due to statistical limitations associated with the sample size and number of variables, it was not possible to explore the potential moderating role of sex in the EF-social functioning relationship. Different indices of EF significantly predicted social symptoms, and sex emerged as an important predictor of social communication and cognition above the effect of EF; therefore future research should employ moderation analyses to understand how EF abilities may impact these social behaviors for males and females. Since previous research suggests that EF is an important mechanism underlying camouflaging behaviors (Hull et al., 2021; Livingston & Happé, 2017) information related to how EF supports or inhibits social behaviors for males and females may have important assessment and treatment implications.

### Limitations and Future Directions

This study explored relations among clinical factors, types of EF impairment, and social symptoms using current measurement approaches among a relatively large sample of well-characterized autistic youth. Nonetheless, there are several limitations. First, given statistical limitations associated with sample size (particularly for females) and number of variables, it was not possible to employ moderation to explore possible sex-based differences in the EF-social communication relationship. Additionally, it was not statistically possible with the given sample to explore the contributions of specific EF scales (e.g., Inhibit, Self-Monitor, Shift) within the BRIEF-2 indices. Future studies with larger sample sizes, increased female representation, and more diverse participants may address these limitations, to elucidate sex-based mechanisms underlying the relationship between EF and social symptoms contributing to divergent symptom presentations. Second, while increased understanding of sex differences in EF and social ASD symptoms may aid understanding of the role of EF in autistic camouflaging, no measures of camouflaging were included in this study. The CAT-Q is a widely used measure validated in autistic adults, but it is not yet validated in youth. Future studies may seek to modify and/or validate the CAT-Q for children and adolescents with ASD. Third, while this study relied on parent-reported symptoms of EF and social communication impairment, observational paradigms that probe for EF in specific social contexts may be helpful in understanding how males and females utilize EF to manage dynamic social encounters with same-age peers. The Contextual Assessment of Social Skills (CASS; Ratto et al., 2011) is a widely used social observational tool where autistic individuals converse with confederates and their social behaviors are rated via a behavioral coding system. Development and implementation of social EF behavioral codes in concert with the established CASS coding system may prove superior for assessing the social impact of EF for autistic males and females. Lastly, it has been shown that sex plays an important role in social communication and cognition above the effect of age, IQ, ASD severity, and EF impairment, future research employing narrow construct measures to study sex differences may focus on these social areas.

## Conclusions

In summary, the findings from this study provide evidence for the contribution of EF to observed social symptoms in ASD. While males and females presented similarly in terms of EF, social impairments, age, and IQ, sex was an important predictor of specific challenges with social communication and cognition. Results suggest there may be sex-based differences in the relationship between EF and social problems for autistic youth, which may have implications for treatment and understanding of the role of camouflaging in social presentations for males and females. Additionally, parents rated females as having more significant impairments in social communication and cognition than males, which may suggest parents rate females’ social symptoms more severely than clinicians or teachers. Further research on the concordance among clinician-, teacher-, and parent-reported social symptoms for autistic females is needed and measures of camouflaging behaviors may elucidate discrepancies across reporters and contexts. It will be important to study sex differences in EF and social ASD symptoms using a multi-measure, multi-informant approach that assesses both broad and narrow social constructs across a variety of contexts.
